# Knowledge Level and Determinants of Neonatal Jaundice: A Cross-Sectional Study in the Effutu Municipality of Ghana

**DOI:** 10.1155/2018/3901505

**Published:** 2018-03-01

**Authors:** Prince Adoba, Richard K. D. Ephraim, Kate Adomakowaah Kontor, Joseph-Josiah Bentsil, Patrick Adu, Maxwell Anderson, Samuel Asamoah Sakyi, Paul Nsiah

**Affiliations:** ^1^Department of Molecular Medicine, School of Medical Sciences, College of Health Sciences, Kwame Nkrumah University of Science and Technology, Kumasi, Ghana; ^2^Department of Medical Laboratory Science, School of Allied Health Sciences, College of Health and Allied Sciences, University of Cape Coast, Cape Coast, Ghana; ^3^Medical OPD, Cape Coast Teaching Hospital, Cape Coast, Ghana; ^4^Laboratory Department, Trauma and Specialist Hospital, Winneba, Ghana; ^5^Department of Chemical Pathology, School of Medical Sciences, College of Health and Allied Sciences, University of Cape Coast, Cape Coast, Ghana

## Abstract

**Background:**

Neonatal jaundice (NNJ) is a major cause of hospital admission during the neonatal period and is associated with significant mortality. This case-control study with cross-sectional design sought to identify the possible factors associated with neonatal jaundice and assess maternal knowledge level of this condition.

**Methods:**

One hundred and fifty (150) neonates comprising 100 with clinically evident jaundice and 50 without jaundice were conveniently recruited from the Trauma and Specialist Hospital in the Effutu Municipality. Blood samples were collected for the determination of serum bilirubin, glucose-6-phosphate dehydrogenase (G6PD), status and blood group (ABO and Rhesus). Well-structured questionnaire was used to collect maternal and neonate sociodemographic and clinical history.

**Results:**

Majority (54%) of neonates developed jaundice within 1–3 days after birth with 10% having it at birth. Duration of labour and neonatal birth weight were associated with neonatal jaundice (*P* < 0.05). G6PD abnormality was found in 11 (12%) of the neonates with jaundice and ABO incompatibility was present in 18%. Neonates delivered by mothers with formal occupation and those who had prolonged duration of labour were significantly more likely to have neonatal jaundice (OR = 4.174, *P* = 0.003; OR = 2.389, *P* = 0.025, resp.). Neonates with low birth weight were also more likely to develop neonatal jaundice (OR = 2.347, *P* = 0.044). Only 17.3% of mothers had heard of neonatal jaundice. School was the major source of information on neonatal jaundice (34.6%). Majority of participants (mothers) did not know that NNJ can cause damage to other organs in the body (90%).

**Conclusion:**

Low neonatal birth weight and prolonged duration of labour are associated with neonatal jaundice. Mothers had inadequate knowledge of neonatal jaundice and its causes.

## 1. Background

Neonatal jaundice (NNJ) is a common condition worldwide occurring in up to 60% of term and 80% of preterm newborns in the first week of life [1, 2]. Newborns show clinical signs which tend to start on the head and face and then spread down the trunk and limbs as a result of high serum levels of bilirubin. Jaundice in newborns is a result of increased release of haemoglobin from breakdown of red cells due to high haemoglobin at birth, as well as due to reduced lifespan of newborn red blood cells (70–80 days) compared to that of adults (90–120 days), and reduced hepatic metabolism of bilirubin due to immature hepatocytes. Most of this newborn hyperbilirubinemia is a natural transition which resolves by the first week of life with maturing of the liver. However, hyperbilirubinemia is also the main reason for hospital admissions and readmission during the neonatal period [3–5]. Hyperbilirubinemia often results in kernicterus with its attendant medical, economic, and social burden on the patients, families, and societies [6, 7].

Several maternal and neonatal risk factors such as preeclampsia, G6PD deficiency, ABO incompatibility, prematurity, birth weight, intrauterine growth retardation, metabolic abnormalities, neonate's gender, birth weight, and nutrition have been identified as risk factors for neonatal jaundice [8, 9].

From the Child Health Outpatient Department of the Korle-Bu Teaching Hospital, Ghana, no day passes without a baby coming in with neonatal jaundice [10]. In a retrospective study conducted by Onyearugha et al. [11] in Nigeria, 35% of neonates managed at a neonatal intensive care unit during a 24-month period were result of jaundice. However, there is scarcity of data on the knowledge and risk factors of neonatal jaundice in Ghana. This study therefore sought to identify the possible factors associated with neonatal jaundice and assess maternal knowledge level of this condition at Winneba in the Effutu Municipality of Ghana.

## 2. Methods

### 2.1. Study Design/Study Site

This hospital-based case-control study with cross-sectional design was conducted from November 2016 to April 2017 at Winneba in the Effutu Municipality of Ghana. The area is known for cohabitation of multiple ethnic groups. The socioeconomic classes range from peasant fisherman and street hawkers to top class civil servants and business executives. The Trauma and Specialist Hospital (TSH) was used for the study. The hospital serves as the central regional hospital providing trauma, orthopedic, and general healthcare to people in and around the region.

### 2.2. Study Population

A total of 150 neonates, comprising 100 with NNJ and 50 without NNJ, were conveniently recruited into the study. All neonates, either outborn or inborn, who presented to the pediatric ward of the hospital were included in the study. Babies who were above 28 days and babies whose parents did not consent to be enrolled were excluded.

### 2.3. Ethical Consideration

Ethical approval was obtained from the University of Cape Coast Institutional Review Board (UCCIRB) and the authorities of the hospital before starting the study. Informed written consent was also sought from the mothers and approval obtained before enrollment into the study.

### 2.4. Collection of Sociodemographic and Clinical Data

Sociodemographic data such as age, marital status, educational level, occupation, and residence and clinical data of neonates and mothers such as gravidity, mode of delivery, duration of labour, ANC visit, bleeding prior to labour, and parity were collected through interview and also from folders using well-structured questionnaire.

### 2.5. Blood Sample Collection

About 4 ml of venous blood samples were drawn from each participant and 2 ml dispensed into a dipotassium ethylenediaminetetraacetic acid (K2-EDTA) tube. The remainder was dispensed into a serum separator tube, allowed to clot, and centrifuged at 1500 rpm for 5–10 mins and the serum used for biochemical analysis.

### 2.6. Biochemical Tests

Serum total and direct bilirubin concentrations were estimated using Pentra C200 chemistry analyzer (34184 Montpellier Cedex 4, France). Indirect serum bilirubin level was calculated by subtracting direct bilirubin from total bilirubin.

### 2.7. Hematological Tests

Neonates' blood group (ABO and Rhesus) and G6PD status were determined using standard protocols [12].

### 2.8. Statistical Analysis

Data were stored in Microsoft Excel and analyzed using Statistical Package for Social Sciences (SPSS) version 16.0 software. Descriptive analysis was performed and the results were expressed as numbers and percentages. Categorical variables were compared using chi-square and quantitative variables by independent *t*-test. Multivariable logistic regression was used to determine the risk factors of neonatal jaundice. *P* < 0.05 was considered statistically significant.

## 3. Results

Table 1 shows sociodemographic characteristics of study participants. The mean age of mothers was 28.3 ± 5.8 years and there was no difference in the ages of those with and those without NNJ. Most of the neonates with NNJ were males (52.7). Most (44%) of the mothers had SHS education with 42% having formal occupation.

The clinical, hematological, and obstetric history of study participants stratified by jaundice status is presented on Table 2. Most of mothers of both NNJ and no NNJ were nulliparous (55% v 56%) and primigravida (45% v 50%). A significant difference was found in proportion of neonates with jaundice and those without jaundice with respect to duration of labour (*P* < 0.05). 15% of mothers with jaundiced neonates experienced vaginal bleeding prior to labour, with 20% being sickling positive. Majority 43% of mothers having neonates with jaundice had prolonged duration of labour compared to the controls (43% v 24%, *P* = 0.023). Most of neonates with jaundice also had low birth weight compared to those without jaundice (34% v 18%, *P* = 0.041).

Majority (54%) of neonates developed jaundice within 1–3 days after birth with 10% having it at birth (Table 3). Mean DB was significantly higher among males than females (13.29 ± 10.42 versus 9.58 ± 3.07, *P* = 0.019). G6PD abnormality was found in 11 (12%) of the neonates with jaundice and ABO blood group incompatibility was present in 18%. No significant difference was found between birth weight, TB, and IDB of male and female neonates with jaundice (*P* > 0.05) (Table 3).

Table 4 presents a correlation of demographic and clinical characteristics of study participants. Maternal age had a positive correlation with gravidity and parity. Gestational age negatively correlated with total bilirubin (*r* = −0.302, *P* = 0.002), direct bilirubin (*r* = −0.239, *P* = 0.017), indirect bilirubin (*r* = −0.296, *P* = 0.003), and birth weight (*r* = −0.393, *P* < 0.001). Total bilirubin had a positive correlation with direct and indirect bilirubin, but a negative correlation with birth weight (*r* = −0.307, *P* = 0.002). Indirect bilirubin also negatively correlated with birth weight (*r* = −0.310, *P* = 0.020).

Neonates delivered by mothers with formal occupation and those who had prolonged duration of labour were significantly more likely to have neonatal jaundice (OR = 4.174, *P* = 0.003; OR = 2.389, *P* = 0.025). Neonates with low birth weight also were significantly more likely to develop neonatal jaundice (OR = 2.347, *P* = 0.044) (Table 5).

Only 17.3% of mothers had heard of neonatal jaundice: 20% of those with babies with NNJ and 12% of those without NNJ. School was the major source of information on neonatal jaundice (34.6%) followed by friends (15.4%), with TV being the least source (7.7%). Majority did not know that NNJ can cause damage to other organs in the body (90%), can be prevented (92.7%), or can be treated (85.3%) (Table 6).

## 4. Discussion

This study sought to identify the possible factors associated with neonatal jaundice and assess maternal knowledge level of this condition at Winneba in the Effutu Municipality of Ghana.

Majority (54%) of neonates developed jaundice within 1–3 days after birth with 10% having it at birth. Birth weight and prolonged duration of labour were associated with neonatal jaundice; mothers had inadequate knowledge of neonatal jaundice.

Most of neonates with jaundice had low birth weight compared to those without jaundice. This is comparable to the findings of a study conducted in Southern Nigeria [13]. Our study also confirmed an earlier observation by Menon and Amanullah [14] and Devi and Vijaykumar [15] which associated neonatal jaundice with low neonatal birth weight in India. This is further buttressed by the finding of neonates with low birth weight being more likely to develop neonatal jaundice in the logistic regression.

Duration of labour was associated with neonatal jaundice, with majority of mothers with jaundiced neonates having prolonged duration of labour compared to the controls. This is similar to the finding of prolonged labour being strongly associated with jaundice in a community-based trial conducted by Scrafford et al. in Nepal [16]. This is most likely due to the clinical relationship between longer duration of labour and cephalohematoma, a known risk factor for severe hyperbilirubinemia [17, 18]. This finding is also supported by the observation of mothers with prolonged duration of labour being more likely to have neonates developing jaundice in our study.

A study conducted in Asia documented ABO incompatibility and G6PD deficiency as the leading causes of neonatal jaundice [19]. G6PD abnormality was found in 11 (12%) of the neonates with jaundice lower than the 25.5% found by Najib et al. [9] in South Iran, but higher than the 4.2% observed by Huang et al. [8] in a case-control study carried out in Taiwan.

ABO incompatibility has been significantly associated with neonatal hyperbilirubinemia [20]. The 18% ABO blood group incompatibility present among neonates with jaundice in our study is higher than the 5.9% reported by Najib et al. [9] in a prospective longitudinal study conducted in Iran, but lower than the 35.5% recorded by Menon and Amanullah [14] in a case-control study conducted in India.

Preterm neonates who have concurrent illnesses and physiologic derangements are more vulnerable to bilirubin neurotoxicity and have been recognized and studied in clinical trials. Bilirubin-related neurotoxicity can result in neonatal death or multisystem acute manifestations and long-term impairments, including irreversible athetoid cerebral palsy (CP) and speech, visuomotor, auditory, and other sensory-processing disabilities [21–23]. Gestational age negatively correlated with total bilirubin, direct bilirubin, indirect bilirubin, and birth weight. This is in line with the finding of significant hyperbilirubinemia among preterm neonates compared to termed neonates in a longitudinal study conducted by Sarici et al. [6] in Turkey. Preterm newborns are prone to developing jaundice due to immaturity of their bilirubin conjugating system, higher rate of haemolysis, increased enterohepatic circulation, and decreased caloric intake [24].

Neonates delivered by mothers with formal occupation were significantly more likely to have neonatal jaundice in this study. This is consistent with the findings of a community-based study conducted in Nigeria by Olusanya et al. [25] in which neonates born to mothers with full time employment were more likely to have jaundice.

Only 17.3% of mothers in this study had heard of neonatal jaundice. This is far lower than the finding of 96% of mothers being aware of neonatal jaundice in a cross-sectional study carried out among expectant mothers in Abia State, southeast Nigeria [11]. In their study, most of the participants got their information from health workers (50%) and friends (26%). However, in our study, school was the major source of information on neonatal jaundice (34.6%) followed by friends, with TV being the least source.

Goodman et al. [26] also found a high level of awareness among mothers (75%) with 74.1% having knowledge of the causes of neonatal jaundice. This is contrary to the finding of majority of our participants (90%) not knowing the cause of neonatal jaundice. Mothers' low knowledge of neonatal jaundice and its causes places them at a very grave risk of ignoring possibly avoidable predisposing factors and even signs that demand immediate management of jaundice in newborns making them develop jaundice and often being presented to healthcare facilities when irreversible neurotoxicity and brain damage might have occurred [26].

Our inability to assess the serum bilirubin, blood group, and G6PD status of neonates with jaundice and other genetic causes of neonatal jaundice (e.g., polymorphisms of UDP-glucuronosyltransferase 1A1 gene) served as a limitation to this study. Also, the study is limited by the small number of neonates without jaundice used.

## 5. Conclusion

Low neonatal birth weight and prolonged duration of labour are associated with neonatal jaundice. Mothers had inadequate knowledge of neonatal jaundice and its causes. Education on the condition and its causes should be intensified especially by healthcare workers during regular antenatal visits. Other causes of neonatal jaundice need to be examined in the routine management of neonates.

## Figures and Tables

**Table 1:** Sociodemographic characteristics of participants.

Parameter	Total	NNJ	No NNJ	*P* value
	(*N* = 150)	(*N* = 100)	(*N* = 50)	
*Mother's age (years)*	28.32 ± 5.81	28.45 ± 5.50	28.06 ± 6.43	0.700
*Mother's age group (years)*				0.092
≤20	14 (9.3)	6 (6.0)	8 (16.0)	
21–30	84 (56.0)	61 (61.0)	23 (46.0)	
31–40	49 (32.7)	32 (32.0)	17 (34.0)	
>40	3 (2.0)	1 (1.0)	2 (4.0)	
*Sex of neonate*				0.817
Male	79 (52.7)	52 (52.0)	27 (54.0)	
Female	71 (47.3)	48 (48.0)	23 (46.0)	
*Mother's occupation*				**<0.001**
None	39 (26.0)	23 (23.0)	16 (32.0)	
Informal	48 (32.0)	23 (23.0)	25 (50.0)	
Formal	63 (42.0)	54 (54.0)	9 (18.0)	
*Residence*				0.318
Town	119 (79.3)	77 (77.0)	42 (84.0)	
Village	31 (20.7)	23 (23.0)	8 (16.0)	
*Marital status*				0.211
Single	55 (36.7)	34 (34.0)	21 (42.0)	
Married	94 (62.7)	66 (66.0)	28 (56.0)	
Divorced	1 (0.7)	0 (0.0)	1 (2.0)	
*Educational level (mother)*				0.176
None	3 (2.0)	2 (2.0)	1 (2.0)	
Primary	2 (1.3)	0 (0.0)	2 (4.0)	
JHS	46 (30.7)	31 (31.0)	15 (30.0)	
SHS	66 (44.0)	48 (48.0)	18 (36.0)	
Tertiary	33 (22.0)	19 (19.0)	14 (28.0)	
*Educational level (father)*				0.658
None	1 (0.7)	1 (1.0)	0 (0.0)	
Primary	0 (0.0)	0 (0.0)	0 (0.0)	
JHS	15 (10.0)	10 (10.0)	5 (10.0)	
SHS	74 (49.3)	52 (52.0)	22 (44.0)	
Tertiary	60 (40.0)	37 (37.0)	23 (46.0)	

NNJ: neonatal jaundice.

**Table 2:** Clinical, haematological, and obstetric history of study participants stratified by jaundice status.

Parameter	Total	NNJ	No NNJ	*P* value
	(*N* = 150)	(*N* = 100)	(*N* = 50)	
*Gravidity*				0.816
Primigravida	70 (46.7)	45 (45.0)	25 (50.0)	
Secundigravida	43 (28.7)	29 (29.0)	14 (28.0)	
Multigravida	37 (24.7)	26 (26.0)	11 (22.0)	
*Parity*				0.737
Nulliparous	83 (55.3)	55 (55.0)	28 (56.0)	
Primiparous	29 (19.3)	18 (18.0)	11 (22.0)	
Multipara	38 (25.3)	27 (27.0)	11 (22.0)	
*Vaginal bleeding*				0.637
No	126 (84.0)	83 (83.0)	43 (43.0)	
Yes	24 (16.0)	17 (17.0)	7 (14.0)	
*Mode of delivery*				0.110
NVD	94 (62.7)	57 (57.0)	37 (74.0)	
NVDA	30 (20.0)	24 (24.0)	6 (12.0)	
CS	26 (17.3)	19 (19.0)	7 (14.0)	
*Duration of labour*				**0.023**
Normal	95 (63.3)	57 (57.0)	38 (76.0)	
Prolonged	55 (36.7)	43 (43.0)	12 (24.0)	
*ANC*				
No	5 (3.3)	4 (4.0)	1 (2.0)	
Yes	145 (96.7)	96 (96.0)	49 (98.0)	
*Place of ANC*				0.318
Clinic	24 (16.1)	17 (17.0)	7 (14.3)	
Hospital	121 (81.2)	79 (79.0)	42 (85.7)	
*Suckling*				0.305
No	13 (8.7)	7 (7.0)	6 (12.0)	
Yes	137 (91.3)	93 (93.0)	44 (88.0)	
*Traditional medicine*				0.134
No	129 (86.0)	89 (89.0)	40 (80.0)	
Yes	21 (14.0)	11 (11.0)	10 (20.0)	
*Mother's sickling status*				0.553
Negative	122 (81.3)	80 (80.0)	42 (84.0)	
Positive	28 (18.7)	20 (20.0)	8 (16.0)	
*Mother's G6PD status*				0.115
Normal	126 (84.0)	80 (80.0)	46 (92.0)	
PD	12 (8.0)	11 (11.0)	1 (2.0)	
FD	12 (8.0)	9 (9.0)	3 (6.0)	
*Birth weight*				**0.041**
Low	43 (28.7)	34 (34.0)	9 (18.0)	
Normal	107 (71.3)	66 (66.0)	41 (82.0)	
Birth weight	2.81 ± 1.72	2.68 ± 0.51	3.06 ± 2.88	0.210
*Gestation*	37.25 ± 1.14	37.50 ± 1.20	37.74 ± 1.01	0.226

NVD: normal vaginal delivery; NVDA: normal vaginal delivery with aid; CS: caesarian section; ANC: antenatal care; PD: partial enzyme defect; FD: full enzyme defect.

**Table 3:** Demographic and clinical characteristics of study participants in relation to sex of neonate.

Parameter	Total	Males	Females	*P* value
	(*N* = 100)	(*N* = 52)	(*N* = 48)	
*Mother's age (years)*	28.45 ± 5.50	27.87 ± 5.73	29.08 ± 5.23	0.271
*Mode of delivery*				0.749
NVD	57 (57.0)	28 (53.8)	29 (60.4)	
NVDA	24 (24.0)	14 (26.9)	10 (20.8)	
CS	19 (19.0)	10 (19.2)	9 (18.8)	
*Duration of labour*				0.507
Normal	57 (57.0)	28 (53.8)	29 (60.4)	
Prolonged	43 (43.0)	24 (46.2)	19 (39.6)	
*Mother's sickling status*				0.484
Negative	80 (80.0)	43 (82.7)	37 (77.1)	
Positive	20 (20.0)	9 (17.3)	11 (22.9)	
*Mother's G6PD status*				0.456
Normal	80 (80.0)	44 (84.6)	36 (75.0)	
PD	11 (11.0)	4 (7.7)	7 (14.6)	
FD	9 (9.0)	4 (7.7)	5 (10.4)	
*Begin of condition*				**0.048**
At birth	10 (10.0)	7 (13.5)	3 (6.2)	
1–3 days	54 (54.0)	22 (42.3)	32 (66.7)	
≥4 days	36 (36.0)	23 (44.2)	13 (27.1)	
*Neonate's G6PD status*				0.511
Normal	88 (88.0)	47 (90.4)	41 (85.4)	
PD	1 (1.0)	0 (0.0)	1 (2.1)	
FD	11 (11.0)	5 (9.6)	6 (12.5)	
*Blood group incompatibility*				0.169
No	82 (82.0)	40 (76.9)	42 (87.5)	
Yes	18 (18.0)	12 (23.1)	6 (12.5)	
*TB (µmol/L)*				0.058
Normal	18 (18.0)	13 (25.0)	5 (10.4)	
High	82 (82.0)	39 (75.0)	43 (89.6)	
*DB (µmol/L)*				0.057
Normal	59 (59.0)	26 (50.0)	33 (68.8)	
High	41 (41.0)	26 (50.0)	15 (31.2)	
*IDB (µmol/L)*				-
High	100 (100.0)	52 (100.0)	48 (100.0)	
*Birth weight*				0.774
Low	34 (34.0)	17 (32.7)	17 (35.4)	
Normal	66 (66.0)	35 (67.3)	31 (64.6)	
*Mean birth weight*	2.68 ± 0.51	2.67 ± 0.53	2.70 ± 0.49	0.705
*Gestation*	37.5 ± 1.20	37.42 ± 1.18	37.58 ± 1.23	0.508
*TB (µmol/L)*	252.44 ± 93.71	260.22 ± 104.5	244.02 ± 80.64	0.390
*DB (µmol/L)*	11.51 ± 7.99	13.29 ± 10.42	9.58 ± 3.07	**0.019**
*IDB (µmol/L)*	240.01 ± 90.54	245.21 ± 100.42	234.37 ± 79.14	0.552

TB: total bilirubin; DB: direct bilirubin; IDB: indirect bilirubin.

**Table 4:** Correlation of demographic and clinical characteristics of study participants.

		Age	Gestation	Gravidity	Parity	TB	DB	IDB	BWt
*Age*									
r		1	0.128	0.715	0.726	0.119	0.099	0.117	0.096
P			0.206	**<0.001**	**<0.001**	0.237	0.326	0.247	0.342
*Gestation*									
r			1	0.065	0.013	−0.302	−0.239	−0.296	−0.393
P				0.522	0.902	**0.002**	**0.017**	**0.003**	**<0.001**
*Gravidity*									
r				1	0.800	0.036	−0.035	0.049	0.070
P					**<0.001**	0.723	0.731	0.627	0.490
*Parity*									
r					1	0.069	−0.059	0.084	−0.024
P						0.496	0.562	0.404	0.810
*TB*									
r						1	0.392	0.990	−0.307
P							**<0.001**	**<0.001**	**0.002**
*DB*									
r							1	0.289	−0.112
P								**0.004**	0.268
*IDB*									
r								1	−0.310
P									**0.020**
*BWt*									
r									1
P									

BWt: birth weight.

**Table 5:** Logistic regression of factors associated with neonatal jaundice (NNJ).

Parameter	OR (95% CI)	*P* value
*Age group (years)*		
≤20	1.500 (0.109–20.675)	0.762
21–30	5.304 (0.459–61.339)	0.182
31–40	3.765 (0.318–44.574)	0.293
>40	Reference	
*Sex*		
Male	0.923 (0.467–1.827)	0.817
Female	Reference	
*Occupation*		
None	Reference	
Informal	0.640 (0.273–1.502)	0.305
Formal	4.174 (1.612–10.807)	**0.003**
*Gravidity*		
Primigravida	Reference	
Secundigravida	1.151 (0.515–2.571)	0.732
Multigravida	1.313 (0.557–3.097)	0.534
*Parity*		
Nulliparous	Reference	
Primiparous	0.833 (0.346–2.003)	0.683
Multipara	1.250 (0.542–2.882)	0.601
*Vaginal bleeding*		
No	Reference	
Yes	1.258 (0.485–3.267)	0.637
*Mode of delivery*		
NVD	Reference	
NVDA	2.596 (0.969–6.957)	0.058
CS	1.762 (0.674–4.603)	0.248
*Duration of labour*		
Normal	Reference	
Prolonged	2.389 (1.117–5.109)	**0.025**
*ANC*		
No	2.042 (0.222–18.764)	0.528
Yes	Reference	
*Suckling*		
No	Reference	
Yes	1.812 (0.575–5.710)	0.310
*Traditional medicine*		
No	Reference	
Yes	0.494 (0.194–1.258)	0.139
*Mother's sickling status*		
Negative	Reference	
Positive	1.313 (0.533–3.231)	0.554
*Mother's G6PD status*		
Normal	Reference	
PD	6.325 (0.791–50.577)	0.082
FD	1.725 (0.444–6.694)	0.431
*Birth weight*		
Low	2.347 (1.022–5.391)	**0.044**
Normal	Reference	

**Table 6:** Mothers' knowledge of neonatal jaundice.

Parameter	Total	NNJ	No NNJ
	(*N* = 150)	(*N* = 100)	(*N* = 50)
*Heard of NNJ*			
No	124 (82.7)	80 (80.0)	44 (88.0)
Yes	26 (17.3)	20 (20.0)	6 (12.0)
*Source*			
Friends	11 (42.3)	10 (50.0)	1 (16.7)
Health facility	4 (15.4)	4 (20.0)	0 (0.0)
School	9 (34.6)	4 (20.0)	5 (83.3)
TV	2 (7.7)	2 (10.0)	0 (0.0)
*Can NNJ cause damage?*			
Do not know	135 (90.0)	89 (89.0)	46 (92.0)
No	5 (3.3)	5 (5.0)	0 (0.0)
Yes	10 (6.7)	6 (6.0)	4 (8.0)
*Can NNJ be prevented?*			
Do not know	139 (92.7)	93 (93.0)	46 (92.0)
No	5 (3.3)	4 (4.0)	1 (2.0)
Yes	6 (4.0)	3 (3.0)	3 (6.0)
*Can NNJ be treated?*			
Do not know	128 (85.3)	83 (83.0)	45 (90.0)
No	0 (0.0)	0 (0.0)	0 (0.0)
Yes	22 (14.7)	17 (17.0)	5 (10.0)

## Data Availability

The datasets used and/or analyzed during the current study are available from the corresponding author on reasonable request.

## Abbreviations

NNJ: Neonatal jaundice
G6PD: Glucose-6-phosphate dehydrogenase.
